# Universal children's day – let's improve current interventions to reduce vertical transmission of HIV now

**DOI:** 10.7448/IAS.17.1.19875

**Published:** 2014-11-20

**Authors:** Mark F Cotton, Helena Rabie

**Affiliations:** Department of Paediatrics and Child Health, Faculty of Medicine and Health Sciences, Stellenbosch University, Stellenbosch, South Africa

## Introduction

By 2013, there was a 40% decline in new paediatric infections compared to 2009. Prevention of mother to child transmission (PMTCT) of HIV continues to reduce the frequency of HIV in infants through wider and earlier implementation of antenatal combination antiretroviral therapy (ART). Post-natally, breastfed infants are now protected from HIV infection through maternal ART and/or infant nevirapine (NVP) [1]. The ultimate aim is the elimination of vertical transmission of HIV.

Should HIV infection occur in infants, the focus is on early detection and treatment. The children with HIV early antiretroviral (CHER) trial established the benefit of initiating ART at a median of seven weeks of age [2,3]. The narrative of the Mississippi child focussed attention on very early diagnosis and therapy. This infant, whose mother was diagnosed as HIV-positive in labour, initiated ART 31 hours after delivery. After ART discontinuation from 15 to 21 months of age due to poor adherence, plasma HIV RNA remained undetectable on standard assays until almost four years of age, with only traces of HIV DNA being found using sophisticated assays [4,5].

## Optimization of antenatal and post-natal maternal HIV diagnosis

The main driver of mother to child transmission is the maternal viral load. Although *in utero* infection has been observed from 12 weeks’ gestation [6], it is likely that the majority occur in the last two months of pregnancy [7]. Therefore, antenatal ART is most effective when commenced by week 20 of pregnancy [8]. Pregnancy is time-limited. Every week of delayed diagnosis increases the risk of vertical transmission. Using data from studies until 2010, Johnson and colleagues calculated that seroconversion late in pregnancy or during breastfeeding contributes 28% of vertical infections, which could be reduced through testing at 32 weeks’ gestation, and at the six-week post-natal visit [9]. Many of these recommendations have now been incorporated in MTCT guidelines, with additional testing in seronegative breastfeeding mothers at three-monthly intervals thereafter recommended [10]. Testing of sexual partners of HIV uninfected pregnant and breastfeeding women may further prevent new acquisition. While the rapid antibody tests are effective, the window period for developing sufficient antibodies for a positive test is between two and eight weeks, with almost 100% being positive after 12 weeks [11]. A new innovation is the point of care virological test, which will substantially increase diagnosis of acute HIV [12]. Dried blood spots offer an appropriate alternative for more resource-constrained settings [13]. Expanded testing should be offered for women where significant risk of acute HIV is identified, for example, those presenting with acute seroconversion illness or in high prevalence settings (Table 1 – see suggested approach to improving maternal diagnosis and ART success).

**Table 1:** Urgent maternal interventions to further reduce HIV PMTCT

Phase	HIV status	Intervention	Rationale	Comments
Antenatal	Unknown	Encourage first clinic visit and initial HIV testing in first trimester	ART most effective when commenced by 20 weeks’ gestation	
	Initial antibody test negative	Repeat HIV testing later in pregnancy and at delivery. If antibody negative, use point of care virological assay Intimate partner testing for HIV uninfected woman	Seroconversion in pregnancy can be detected and infants identified for triple ARV post-exposure prophylaxis. Stratification of tests allows rational usage of antibody and virological testing Preventing maternal seroconversion	Initiate ART rapidly. Raltegravir can be added to more rapidly reduce viral load
	HIV +	Check ART adherence through standard viral load assays at 32 weeks’ gestation and ensure no missed pharmacy visits	Standard viral load assay often easily available in many settings. Pharmacy visits easy to measure	Standard test can be replaced by point of care assay if standard test logistically difficult
During Delivery	HIV+	Point of care virological test if assay at 32 weeks’ gestation above detectable limits	Identifies infants for ART prophylaxis and early diagnosis	
	Previously HIV negative or unknown	Point of care antibody test antibody test first; if negative, use point of care virological assay	As above	

## Infant post-exposure prophylaxis when there is a high risk of vertical transmission

For new HIV diagnosis in late pregnancy or labour, there is a possibility of reducing risk of vertical transmission through enhanced post-exposure prophylaxis. A randomized study of post-exposure prophylaxis for infants born to women from high prevalence settings where HIV was diagnosed late in pregnancy and who received no antenatal ARVs, has contributed many insights. The main objective was to determine the best post-exposure regimen for infants at extremely high risk for vertical HIV infection. Two antiretrovirals (ARVs) (zidovudine [ZDV] for six weeks plus three doses of NVP in the first week or three ARVs (ZDV, lamivudine [LMV] and nelfinavir) for two weeks performed equally well and were superior to ZDV given for six weeks in formula-fed infants [14]. Given the superior efficacy of three ARVS compared to two ARVs for treatment, many would have hoped that the three-drug regimen would be better. Reasons for equivalence include inability to achieve therapeutic exposure for nelfinavir in 46% of infants, although nearly all had trough levels above 0.05 µg/ml, 10 times the upper limit of nelfinavir IC50 for wild type HIV [15]. Also, the standard duration of post-exposure prevention is four weeks, rather than the two weeks used in this study [16]. Post-exposure prophylaxis for 28 days rather than a shorter period is supported by rhesus macaque studies after simian immunodeficiency virus infection [17,18]. A recent cohort analysis from Europe showed that a three-ARV regimen for 28 days in post-exposure prophylaxis is being used more frequently in circumstances where there is a high risk of vertical transmission [19]. This strategy is being widely adopted subsequent to the report of the Mississippi baby [4] and experience in Canada [20].

## Early infant diagnosis of HIV

In the post-exposure prophylaxis study by Nielsen-Saines *et al*., a polymerase chain reaction (PCR) test on the first day of life showed *in utero* infection in 5.7% of infants, illustrating the importance of an immediate PCR when the risk of transmission is high. There is a growing concern that both the accuracy of diagnostic testing and relying on a single test in the presence of ARV exposure are insufficient, leading to a false sense of security. In one report, a PCR at 4–6 weeks of age in formula-fed infants missed 32% of infections found at three months of age. Other studies have confirmed the inaccuracy of a single PCR at 4–6 weeks of age [21,22]. Point of care virological testing may be useful on the first day of life particularly where there is a high transmission risk but is not essential provided that at least two blood specimens are drawn for PCR in the first few days of life and triple ARV prophylaxis is initiated as soon as possible after birth. The latter should then be continued if the infant is HIV-infected. Also, for breastfed infants regular virological testing of the infant is essential until the infant is fully weaned (see Table 2).

**Table 2:** Urgent measures to increase early diagnosis, prevention and treatment in HIV-exposed infants

Timing	Intervention	Comment
At birth	Commence post-exposure prophylaxis using three ARVs for 4 weeks	Begin as soon as possible and take precedence over timing of diagnostic tests. Convert to continued ART for a positive early test
	2 separate blood draws for diagnostic PCR tests or point of care virology test plus confirmatory PCR	Confirmation of HIV status essential
4–6 weeks	Repeat diagnostic PCR	
12 weeks	Repeat diagnostic PCR if negative at 4–6 weeks	Delayed positive test possible
For continued breastfeeding	Repeat diagnostic PCR every 3 months Rapid antibody test or virological point of care test can be instituted at 9 months of age	This should continue until 6 weeks after fully weaned.A positive rapid antibody test at 9 months may reflect slow disappearance of maternal antibody and requires a diagnostic PCR or point of care virology test

## Conclusions

PMTCT approaches in high prevalence settings require urgent adaptation to improve early infant diagnosis for the prevention and management of HIV.
